# The Interplay between Immune System and Microbiota in Diabetes

**DOI:** 10.1155/2019/9367404

**Published:** 2019-12-30

**Authors:** Simona Moffa, Teresa Mezza, Chiara M. A. Cefalo, Francesca Cinti, Flavia Impronta, Gian Pio Sorice, Antonio Santoro, Gianfranco Di Giuseppe, Alfredo Pontecorvi, Andrea Giaccari

**Affiliations:** Centro Malattie Endocrine e Metaboliche, UOC Endocrinologia e Diabetologia, Fondazione Policlinico Universitario A. Gemelli IRCCS and Istituto Patologia Speciale Medica e Semeiotica Medica, Università Cattolica del Sacro Cuore, Rome, Italy

## Abstract

Diabetes is not a single and homogeneous disease, but a cluster of metabolic diseases characterized by the common feature of hyperglycemia. The pathogenesis of type 1 diabetes (T1D) and type 2 diabetes (T2D) (and all other intermediate forms of diabetes) involves the immune system, in terms of inflammation and autoimmunity. The past decades have seen an increase in all types of diabetes, accompanied by changes in eating habits and consequently a structural evolution of gut microbiota. It is likely that all these events could be related and that gut microbiota alterations might be involved in the immunomodulation of diabetes. Thus, gut microbiota seems to have a direct, even causative role in mediating connections between the environment, food intake, and chronic disease. As many conditions that increase the risk of diabetes modulate gut microbiota composition, it is likely that immune-mediated reactions, induced by alterations in the composition of the microbiota, can act as facilitators for the onset of diabetes in predisposed subjects. In this review, we summarize recent evidence in the field of gut microbiota and the role of the latter in modulating the immune reactions involved in the pathogenesis of diabetes.

## 1. Introduction

Diabetes can be described as a cluster of metabolic diseases characterized by the common feature of hyperglycemia. However, it is not a single and homogeneous disease and is therefore difficult to classify.

In the past, it was categorized on the basis of age at diagnosis and the need for insulin therapy. The latest pathogenetic [1] classification identifies four forms of diabetes; in particular, the subdivision into type 1 (T1D) and type 2 (T2D) diabetes was introduced to replace insulin-dependent and noninsulin-dependent diabetes.

T1D is the most common metabolic disorder in children and young adults, and is due to a progressive autoimmune or idiopathic *β*-cell destruction, with the end result of absolute insulin deficiency. It is a multifactorial disease, in which a genetic predisposition, combined with a triggering event, initiates the activation of self-reactive lymphocytes. Although in the early stages the disease is clinically silent, it is already possible to detect autoantibodies directed against *β*-cell antigens. Several factors have been hypothesized to contribute to T1D onset, including chemicals, viruses, commensal bacteria, and diet. T2D, on the other hand, most commonly occurs in adulthood, against a background of obesity and insulin resistance. It is characterized by an initial phase of compensatory hyperinsulinemia, creating an overload for pancreatic *β*-cells, leading to a progressive loss of insulin secretive function, and consequently to hyperglycemia.

However, several studies have shown that this subdivision does not accurately describe some intermediate forms of diabetes, with overlapping features [2]. It is increasingly common to see obese young people with metabolic characteristics of T2D with autoantibodies for *β*-cells typical of T1D now defined as “double diabetes” or type 1.5 diabetes [3]. Moreover, there is another well-known type of diabetes, called Latent Autoimmune Diabetes in Adults (LADA), which shares mechanisms belonging to the two abovementioned diseases: a progressive reduction in insulin secretion due to autoimmune destruction of *β*-cells and, although to a lesser extent than in T2D, insulin resistance.

Over the past decades, there has been an increase in all forms of diabetes, accompanied by changes in eating habits and consequently a structural evolution of gut microbiota [4]. It is likely that all these events could be related and that gut microbiota alterations might be involved in the immunomodulation of diabetes.

Immunomediated pathogenesis is a common feature of almost all forms of diabetes, in terms of inflammation and/or autoimmunity. On the other hand, alterations of gut microbiota seem to be linked to several immune-driven inflammatory diseases [5–7]. These observations have led researchers to hypothesize a possible link between diabetes onset and gut microbiota alterations.

Against this complex background, recent studies have shown that the development of diabetes is closely related to alterations of gut microbiota, an important “organ” consisting of bacteria, viruses, protozoa, and fungi living in the gastroenteric tube [8]. The microbiota provides protection against pathogenic microbes by maintaining local intestinal integrity and regulating intestinal barrier permeability.

All this is possible thanks to a symbiotic relationship favored by the balance between gut microbiota, intestinal epithelial cells, and the mucosal immune system [9]. The disruption of this equilibrium, called dysbiosis, seems to be involved not only in the pathogenesis of several intestinal diseases, such as inflammatory bowel disease (IBD) [10], celiac disease, irritable bowel syndrome (IBS), and colorectal cancer [11], but also in metabolic diseases including obesity, metabolic syndrome, and diabetes [12, 13]. Recently, intestinal microbiota composition has been shown to play a role in obesity [14] and diabetes [15], but the exact molecular mechanisms through which a given intestinal microbiota induces metabolic diseases still need to be clarified. In the case of T2D, increased energy harvesting and the triggering of a low-grade inflammatory status in insulin resistance and obesity [16] are two of the possible mechanisms involved. Therefore, recent studies also suggest that gut microbiota contributes to the risk of developing T1D in genetically predisposed individuals; indeed, environmental factors that may affect the risk of developing T1D, including birth delivery mode [17], diet in early life [18], and possibly the use of antibiotics [19], are all related to the intestine and its microbiota.

Thus, gut microbiota seems to have a direct, even causative role in mediating connections between the environment, food intake, and chronic disease. Whereas many conditions that increase the risk of diabetes modulate gut microbiota composition, it is likely that immune-mediated reactions induced by alterations in the composition of the microbiota can act as facilitators of the onset of diabetes in predisposed subjects.

In this review, we will summarize the recent evidence in the field of gut microbiota and the role of the latter in modulating immune reactions involved in the pathogenesis of diabetes.

## 2. Gut Microbiota Composition in Diabetes

Intestinal microbiota is made up of five dominant bacterial phyla: *Firmicutes*, *Bacteroidetes*, *Actinobacteria*, *Proteobacteria*, and *Verrucomicrobia* [20]. In particular, *Bacteroidetes* and *Firmicutes* are the main bacterial phyla known to be correlated with obesity and T2D. The *Firmicutes* phylum is composed of *Ruminococcus*, *Clostridium*, *Lactobacillus*, and butyrate-producing bacteria, while the *Bacteroidetes* phylum consists of *Bacteroides*, *Prevotella*, and *Xylanibacter* [20].

Human and animal studies have been used to demonstrate that gut microbiota composition is altered in diabetes. Comparing the gut microbiota of lean mice and mice with diet-induced obesity, some authors found an increase in the abundance of *Firmicutes* associated with diet-induced obesity [21]. These observations were supported by the identification of an increase in the *Firmicutes/Bacteroidetes* ratio in ob/ob mice and in mice fed a high-fat diet compared with lean mice. Furthermore, this increase was more significant in the high-fat diet-fed mice than in the ob/ob mice [22].

Other studies have also demonstrated a strong connection between T2D and changes in the composition of gut microbiota. A study conducted on diabetic patients compared to nondiabetic controls showed that the proportions of phylum *Firmicutes* and class *Clostridia* were significantly reduced in the diabetic group compared to the control group, while there was a greater quantity of *Bacteroidetes* and *Proteobacteria*. Consequently, the ratios of *Bacteroidetes* to *Firmicutes* were found to be significantly and positively correlated with reduced glucose tolerance [15].

In humans, however, there are still doubts as to whether the state of intestinal microbiota is the consequence or the cause of the altered metabolic condition. To clarify this, studies using germ-free mice have demonstrated the central role of intestinal microbiota in triggering metabolic impairments, even though it remains to be demonstrated whether genetic background can influence the development of a specific microbiota.

Diet is one of the main determinants of intestinal microbiota composition and an extremely important causal factor in the development of T2D. Turnbaugh et al., for example, have shown that microbiome structure is rapidly altered in response to a switch from a low-fat, plant polysaccharide-rich diet to a high-fat, high-sugar “Western” diet [23].

In the last decades, human food habits have changed, with fats being preferred over fibers; thus, gut microbiota has changed in response to the new feeding habits. It has therefore been hypothesized that the diabetes epidemic could be related to the structural change of gut microbiota.

Studies have found that in T1D there is an imbalance in intestinal microbiota; thus, children with T1D showed higher levels of *Bacteriodetes* than controls, who instead had higher levels of *Prevotellla* [24]. Other studies have found a reduction in beneficial anaerobic bacteria in children with T1D and an increase in *Enterobacteriaceae*, and proposed this as a possible immune trigger for T1D onset [25]. A Finnish study that evaluated children at high genetic risk for T1D, following them from birth to 2.2 years of age, showed that the species *Bacteroides dorei* and *Bacteroides vulgatus* were found in greater numbers in T1D cases compared to controls prior to seroconversion, suggesting that early changes in microbiota composition could be useful in predicting T1D autoimmunity in genetically susceptible infants [26].

Diabetes-related alterations in gut microbiota composition have also been associated with exposure to xenobiotics, such as heavy metals, persistent organic pollutants (POPs), and organophosphate. In the last decades, there has been a massive production and release of toxic chemicals affecting the entire globe. Many of these chemicals interfere with the endocrine system altering hormone production, release, transport, and activities and are known as endocrine-disrupting chemicals (EDCs) [27]. EDCs enter the human body mainly through the mouth, and gut microbiota plays a central role in their metabolism, thus contributing to obesity and diabetes [28]. Among heavy metals, arsenic exposure in mice seems to affect gut microbiota composition, not altering *Bacteriodetes* but decreasing several species of *Firmicutes* (*Eubacterium*, *Faecalibacterium*, and *Roseburia*), leading to metabolomic changes in gluconeogenesis, adipogenesis, lipogenesis, and inflammation [29]. Regarding POPs, exposure to 2,3,7,8-tetrachlorodibenzofuran (TCDF) affected gut microbial structure, with a decrease in the *Firmicutes*/*Bacteriodetes* ratio. TCDF also increased levels of Flavobacteriia and *Butyrivibrio* spp. and decreased Clostridia and *Oscillobacter* spp. resulting in an increase in bile acid metabolites. Increased levels of SCFAs were also observed in fecal and cecal contents of TCDF-exposed mice and may be responsible for altered hepatic lipogenesis, gluconeogenesis, and glycogenolysis [30]. Organophosphates (OPs) are chemical substances used in insecticides, herbicides, etc., which inhibit acetylcholine esterase; animal studies have shown that prolonged intake of the OP insecticide monocrotophos induces hyperglycemia, dyslipidemia, cardiac oxidative stress, and myocardial infarction in rats [31] An altered hepatic gluconeogenesis mediated by OP-degrading gut microbiota has been demonstrated to be the key mechanism underlying OP-induced hyperglycemia. [32]

Hence, structural alterations of gut microbiota seem to characterize all forms of diabetes. In fact, these compositional alterations may play a role in both inducing the onset of autoimmune diabetes in young predisposed subjects and in speeding up the process of *β*-cell failure in obese/insulin-resistant subjects.

## 3. Gut Microbiota in Immunopathogenesis of Diabetes

The microbes making up the gut microbiome utilize nutrients and produce metabolites which are able to influence metabolism, leading to obesity, insulin resistance, and diabetes. For example, short-chain fatty acids (SCFAs) are produced by fermentation of dietary fibers; these molecules act both as energy substrates used by colonocytes and the host, and as ligands for G-protein-coupled receptors (GPCRs) [33]. These GPCRs, under SCFA stimulation, induce peptide YY (PYY) production, which can modulate intestinal motility and nutrient absorption. A study of murine germ-free and cocolonized Gpr41-/- and +/+ littermates showed that Gpr41-deficiency is associated with reduced expression of PYY, increased intestinal transit rate, and reduced harvest of energy from the diet. These results reveal that Gpr41 is a regulator of host energy balance through effects that are dependent upon the gut microbiota [34].

The obese microbiome has an increased capacity to harvest energy from the diet, and this feature is genetically transmissible: some researchers have shown that colonization of germ-free mice with an obese microbiota led to a greater increase in total body fat than colonization with lean microbiota [21]. Furthermore, germ-free (GF) animals are protected against obesity after consuming a Western-style, high-fat, sugar-rich diet. Two complementary but independent mechanisms that result in increased fatty acid metabolism are involved here: elevated levels of fasting-induced adipose factor (Fiaf), a circulating lipoprotein lipase inhibitor which induces peroxisomal proliferator-activated receptor coactivator (Pgc-1*α*), and increased phosphorylated AMP-activated protein kinase (AMPK) activity [35]. These findings suggest that manipulation of the gut microbiota may impact on muscle activity, regulating fatty acid oxidation. Thus, the host energy metabolism may be protected against a high-calorie westernized diet. Furthermore, exercise intervention has been shown to directly affect gut microbiota composition; germ-free mice exhibited worse exercise performance compared to mice colonized by a single bacterial species, while mice colonized by multiple nonharmful bacteria displayed the best exercise performance [36]. Moreover, another study showed that in obese mice, even under high-fat diet conditions, exercise protects gut microbiota by reducing inflammatory markers such as cyclooxygenase 2 (Cox-2) in both the proximal and distal gut [37]. Human studies have also revealed the importance of physical exercise in modulating gut microbiota in order to prevent/ameliorate metabolic diseases. Estaki et al., after normalizing BMI, diet, and age, analyzed fecal microbiota and fecal SCFAs in 39 healthy subjects and found that a higher fitness level correlated with gut microbiome diversity. Furthermore, increased production of butyrate, a marker of gut health, and increased abundance of butyrate-producing species were found in individuals with greater levels of aerobic fitness [38]. Another study showed that in T2D patients, a six-month endurance, resistance, and flexibility training program decreased intestinal mycetes overgrowth, gut permeability, and systemic inflammation, resulting in improved glycemia and functional and anthropometric variables [39].

Obesity and insulin resistance, leading to T2D, are characterized by low-grade inflammation, consequent to a morbid activation of the immune system. Lipopolysaccharides (LPS), which are components of the outer membrane of Gram-negative bacteria, with their high inflammatogenic properties, were thought to be the precipitators of the inflammatory processes leading to obesity and insulin resistance [40]. LPS are able to cross the intestinal epithelial barrier either via leaky intestinal tight junctions or carried by chylomicrons [41]. Once they reach systemic circulation, LPS bind the plasma LPS-binding protein (LBP), which activates the receptor protein CD14 located in the plasma membrane of macrophages. This complex is able to bind Toll-like receptor 4 (TLR4) on the membrane of macrophages, triggering the synthesis of several inflammatory effectors, such as nuclear factor *κ*B (NF-*κ*B) and activator protein 1 (AP-1) [42]. This has been confirmed in mice with deletions of the LPS receptor TLR4, or part of the TLR4 machinery such as CD14, that showed attenuated inflammatory response and increased glucose transport; in addition, TLR4 inactivation blunted insulin resistance induced by LPS in differentiated adipocytes [43]. The involvement of gut microbiota has been further demonstrated since, in a study by Cani et al., chronic antibiotic treatment reduced metabolic endotoxemia and the cecal content of LPS in both high-fat-fed and ob/ob mice. This effect was correlated with reduced glucose tolerance and body weight gain. Furthermore, a high-fat diet was shown to greatly increase intestinal permeability and reduce the expression of genes coding for proteins of the tight junctions [44].

Thus, gut microbiota can affect intestinal mucosal permeability, leading to an increased absorption of exogenous antigens [45]. Furthermore, some microbial toxins have been reported to directly impair pancreatic *β*-cell function [46], which could be one of the mechanisms underlying autoimmune diabetes. In fact, in mice models, the injection of *Streptomyces* toxin and bafilomycin A1 resulted in smaller islets and reduced the entire pancreatic *β*-cell mass, concurrently impairing glucose tolerance [46]. Moreover, Lee et al. have also demonstrated that gut barrier disruption induced by *C. rodentium* infection accelerated insulitis in NOD mice [47]. Therefore, innate immune cells are directly involved in linking gut microbiota and diabetes pathogenesis. Any alteration in the communication between innate immunity components and gut microbiota may lead to diabetes onset, as explained above.

Several studies have investigated the role of the innate immune system at the intestinal level and also in T1D pathogenesis. Deletion of the innate immune adaptor myeloid differentiation primary response gene 88 (MyD88) in a NOD mouse model of T1D provided microbiota-dependent protection from the disease: MyD88-negative mice in germ-free (GF) but not in specific pathogen-free conditions develop the disease. The same authors also found that colonization of GF mice with a variety of intestinal bacteria reduced the occurrence of T1D in MyD88-negative but not wild-type NOD mice, favoring the balanced signal hypothesis: i.e., that both inflammatory and regulatory responses are induced by the microbiota and that TLR4-mediated Trif signaling causes a tolerizing immune response, which protects against T1D development [48].

Going back to talk about T2D, another mechanism involved in dysbiosis-induced immunopathogenesis of obesity and T2D is that of alterations in T helper 17/regulatory T cell (Th17/Treg) balance. Th17 and Treg cells are two CD4^+^ T helper cells; Treg cells regulate and control immune tolerance in healthy individuals, while Th17 cells mainly produce IL-17. Th17 cells have been implicated in the control of adipogenesis and glucose homeostasis in obesity [49]. In a recent study, intestinal ROR*γ*t^+^ IL-17^+^CD4^+^ T-cells were shown to participate in energy metabolism in mice, and specifically, a reduction of ROR*γ*t^+^ and IL-17-producing CD4^+^ T-cells contributed to the development of insulin resistance [50]. This observation supports the role of the intestinal Th17 lineage in the regulation of insulin sensitivity.

Several studies have shown that gut microbiota alterations are associated with abnormalities of the mucosal immune system, likely involved in the autoimmunity underlying T1D. In a murine study, the transfer of intestinal *Lactobacillus johnsonii* N 6.2 from diabetes-resistant biobreeding rats to diabetes-prone biobreeding (BBDP) rats resulted in a delay in disease pathogenesis through a mechanism that might involve the upregulation of Th17 cells [51]. These findings concur with those of another study, in which protection from the disease, probably mediated by the upregulation of intestinal T helper 17 cells, was observed after segmented filamentous bacteria were naturally transmitted to NOD mice [52]. These data seem to prove that bacteria provide protection against disease in both BBDP and NOD murine models.

Th17 cells are involved in different ways in T1D pathogenesis. In a spontaneous autoimmune diabetes model, IL-17A and IL-17F expressions in islets are related to insulitis in NOD mice. However, islet antigen-specific Th17 cells need to be transformed into Th1-like cells to induce diabetes [53]. Furthermore, a recent study has revealed that the exposure of nonobese diabetic NOD mice to acidified water was able to delay T1D onset: NOD mice exposed to neutral water, in fact, were more predisposed to the development of diabetes, while exhibiting a decrease in *Firmicutes* and an increase in *Bacteroidetes*, *Actinobacteria*, and *Proteobacteria*. They also had lower levels of Foxp3 expression in CD4(+)Foxp3(+) cells, as well as decreased CD4(+)IL-17(+) cells, and a lower ratio of IL-17/IFN-*γ* CD4^+^ T-cells, indicating that a change in liquid acidity dramatically alters the intestinal microbiome, the presence of protective Th17 and Treg cells, and the incidence of diabetes [54]. Moreover, in NOD mice, a Th17/Treg imbalance compromises the ability of Treg cells to suppress self-reactive effector T-cells and to impede the destruction of pancreatic islets, which may potentially induce or aggravate T1D [55].

The balance of Th1/Th2 lymphoid cells also seems to play a role in diabetes onset mediated by gut microbiota. Th1/Th2 lymphoid cells are CD4^+^ T-cells, whose differentiation into Th1 or Th2 depends on stimulation by IFN-*γ* and IL-4, respectively. Several studies have shown that gut microbiota and its metabolites can modulate the equilibrium of Th1/Th2 cells in the intestinal tract. For example, the polysaccharide A produced by an anaerobic gram-negative bacterium *Bacteroides fragilis* could promote the expression of proinflammatory cytokines, such as IL-12 and p40, leading to Th1 activation [56]. Furthermore, Pam3 of gram-positive bacteria can activate IFN-*γ* production, in turn inducing the differentiation of Th1 cells [57]. Commensal A4 bacteria belonging to the Lachnospiraceae family produce an immunodominant microbiota CBir1 antigen by inducing TGF-*β* production by dendritic cells [58].

IL-12 is also the primary immunoregulatory factor secreted by Th1 cells and plays a key role in the pathogenesis of diabetes. IL-12 is able to bind to IL-12 receptors on pancreatic *β*-cells and activate proinflammatory cytokines (IL-1*β*, TNF-*α*, and IFN-*γ*), inducing their apoptosis via the STAT4 signaling pathway [59]. In addition, IL-12 is involved in complications of T2D; Ali et al. recently determined that the disruption of IL-12 promotes angiogenesis and increases blood flow in obese type 2 diabetic mice by an endothelial nitric oxide synthase/Akt/vascular endothelial growth factor receptor 2/oxidative stress-inflammation-dependent mechanism [60].

The nucleotide-binding oligomerization domain-containing protein 2 (Nod2) has been identified as a key factor for T1D susceptibility; Nod2^−/−^NOD mice had different gut microbiota compared to Nod2^+/+^NOD mice and were protected from diabetes, but only when kept separate from Nod2^+/+^NOD mice, suggesting that T1D susceptibility in Nod2^−/−^NOD mice is dependent on the alteration of gut microbiota. In fact, colonizing germ-free NOD mice with Nod2^−/−^NOD microbiota significantly reduced the number of cells secreting proinflammatory cytokines but increased T-regulatory cells [61].

A recent study also evaluated the ability of human gut microbiota to delay the onset of T1D when transferred into germ-free NOD mice; diabetes onset was significantly delayed in all bacteriome humanized colonies vs. germ-free NOD mice, but the pace of beta cell loss was not transferable to the mouse model [62].

Physical exercise also seems to play a role in the immunomodulation of gut microbiota involved in T1D pathogenesis. A recent study revealed that NOD mice subjected to moderate intensity exercise benefited from glucose-lowering effects in the late stages of diabetes, while control sedentary NOD mice showed larger infiltrates at the end of the 12-week study. These findings suggest that exercise could promote a beneficial immune-modulation in T1D [63].

Thus, gut microbiota can modulate both innate and adaptive immunity, resulting in conditions which facilitate diabetes onset. This is possible due to gut microbiota's ability to affect glucose metabolism under predisposing conditions such as obesity and metabolic syndrome, and in conditions at risk of autoimmunity.

## 4. Gut Microbiota as a Novel Therapeutic Target for Prevention and Treatment of Diabetes

### 4.1. Probiotics

The aforementioned evidence has raised interest in targeting gut microbiota as an effective strategy to prevent and manage diabetes.

Probiotics are living microorganisms that can be ingested either alone or with food, conferring benefits to their host [64]. Yadav et al. showed that a probiotic dahi-supplemented diet, containing *Lactobacillus acidophilus* and *Lactobacillus casei*, significantly delayed the onset of glucose intolerance, hyperglycemia, hyperinsulinemia, dyslipidemia, and oxidative stress in high fructose-fed diabetic rats, thus lowering the risk of diabetes and its complications [65]. Other authors have investigated the effects of probiotic yogurt containing *Lactobacillus acidophilus* La5 and *Bifidobacterium lactis* Bb12 and conventional yogurt on blood glucose and antioxidant status in type 2 diabetic patients; the probiotic yogurt improved fasting plasma glucose values and HbA1c and antioxidant status in diabetic subjects [66]. Moreover, daily consumption of 200 ml of a shake containing 4 × 108 CFU/100 ml of *Lactobacillus acidophilus*, 4 × 108 CFU/100 ml of *Bifidobacterium bifidum*, and 1 g/100 ml of fructooligosaccharides decreased blood glucose in T2D individuals. Recently, a growing interest for *Akkermansia muciniphila* developed since it seems to ameliorate gut permeability, obesity, and glucose tolerance [67, 68]. A recent randomized, double-blind, placebo-controlled study first evaluated metabolic effects of *Akkermansia muciniphila* administration in overweight/obese insulin-resistant humans. It showed a positive effect on insulin sensitivity and total cholesterol, and it turned out to be safe and well tolerated [69].

Several studies have also investigated fecal transplants as a therapeutic strategy. An animal study showed that the bacterial transfer from MyD88-deficient NOD mice, which are protected from T1D development, reduced insulitis and significantly delayed the onset of diabetes. Moreover, after the oral transfer of fecal bacteria over 3 weeks, the composition of the gut microbiota was stably altered, as it showed an increase in *Lachnospiraceae* and *Clostridiaceae* and a decrease in *Lactobacillaceae* [70]. Furthermore, the TEDDY study, a prospective cohort study that followed children at high risk for autoimmune diabetes, observed a reduction in the risk of islet autoimmunity in children who had received probiotics before or at the age of 27 days compared with those who had first received probiotics after 27 days or not at all [71].

A number of studies in humans have explored the effects of infusing intestinal microbiota from lean donors to male recipients with metabolic syndrome. Vrieze et al. demonstrated that six weeks after infusion of microbiota from lean donors, insulin sensitivity of recipients and levels of butyrate-producing intestinal microbiota increased [72], and they concluded that butyrate-producing bacteria prevent translocation of endotoxic compounds derived from gut microbiota, one of the factors driving insulin resistance. Similarly, another study has suggested that the butyrate synthesizing microbiota could improve insulin sensitivity through signaling pathways and direct effects on glucose metabolism [73]. Thus, intestinal microbiota transplantation, especially *F. prausnitzii*, from a normal individual to a diabetic one, seems to be able to synthesize abundant quantities of butyrate, which stabilizes the leaky gut and inhibits downstream proinflammatory mechanisms.

Probiotic supplementation or microbiota transplantation are two promising novel therapeutic strategies that could be used to prevent or treat diabetes by modulating the host's preexisting microbiota.

### 4.2. Prebiotics

Prebiotics are defined as food able to induce a selective growth and/or activity of one or a limited number of microbial genus(era)/species in the gut microbiota that confer(s) health benefits to the host [74]. Prebiotics are mainly inulin, fructooligosaccharides, galactooligosaccharides, and lactulose [75]. Kim et al. conducted a trial to evaluate the effect on glucose, lipid metabolism, and fecal microbiota composition of a one-month strict vegetarian diet in six obese subjects with T2D and/or hypertension. A strict vegetarian diet reduced body weight and the concentration of triglycerides, total cholesterol, low-density lipoprotein cholesterol, and HbA1c and improved fasting glucose and postprandial glucose levels. In addition, it determined compositional changes in gut microbiota, such as a reduced ratio of *Firmicutes* to *Bacteroidetes* and an increase in the species of *Bacteroides fragilis* and *Clostridium*, which decreased intestinal inflammation and SCFA levels [76]. Another randomized, placebo-controlled trial assessed the effects of the administration of high performance inulin on glycemic status and lipid profile in women with T2D. Forty-nine subjects were randomized to receive 10 g/d inulin or 10 g/d maltodextrin for 8 weeks. The inulin-treated group showed a significant reduction in fasting plasma glucose, HbA1c, total cholesterol, and triglycerides and an increase in HDL-C [77]. Furthermore, a cross-sectional study by Beretta et al. revealed that a higher fiber intake in T1D is also associated with lower systolic and diastolic blood pressure [78].

Several antidiabetic drugs act as prebiotics, inducing changes in gut microbiota composition. Metformin, which represents the first-line therapy in T2D management, is able to positively modulate gut microbiota composition, both in humans and animals [79], contrasting the effects of a high-fat diet [80]. The exact mechanism by which metformin acts on gut microbiota composition is still unknown, but it seems to decrease the abundance of *Intestinibacter* [81] while increasing butyrate production [82]. Some studies have shown that it is able to inhibit bacterial complex I and also has an antimalarial function [83]. The degree to which gut microbiota is altered by metformin depends on host factors, such as the dosage used, the oral availability of the drug, and personal variability in absorption.

Glucagon-Like Peptide-1 Receptor Agonists (GLP-1RAs), another class of widely used antidiabetic drugs, also seem to have a role in modulating gut microbiota. Wang et al. showed that in mice treated with liraglutide, compared to saxagliptin, a lean-related gut microbiota profile was consistent with the loss of body weight [84]. Other studies have shown that liraglutide prevents diabetes onset in male rats and that this effect seems to be correlated with structural changes in gut microbiota, specifically an increase in SCFA-producing bacteria (*Bacteroides*, *Lachnospiraceae*, and *Bifidobacterium*) [85].

## 5. Conclusions

It is well known that innumerable pathogenic mechanisms are involved in all forms of diabetes.

**Figure 1:**
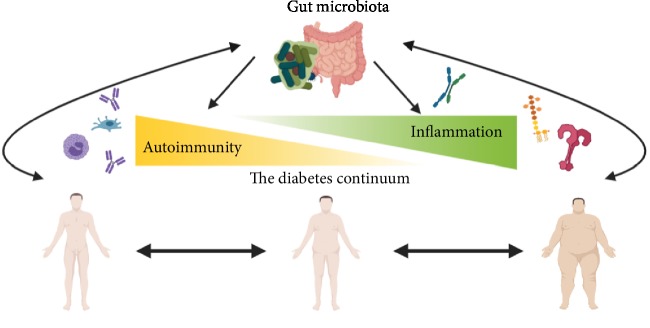
Gut microbiota alterations predisposing both autoimmunity and inflammation facilitate the appearance of all forms of diabetes: from T1D to T2D passing through LADA and other intermediate forms of diabetes. Likewise, diabetes itself can modulate gut microbiota, inducing structural and functional alterations that contribute to the disease.

Considering the data presented above, it appears evident that structural and functional alterations of intestinal microbiota are present not only in overt diabetes but also in conditions which predispose towards diabetes, such as obesity, metabolic syndrome, and the presence of antibodies associated with immune-mediated diabetes. Moreover, many studies (summarized in Table 1) have shown that these alterations trigger an innate and adaptive immune response which finally leads to overt diabetes. Although not conclusive, the evidence points towards the microbiota inflammation/autoimmunity diabetes hypothesis. It is likely that microbiota alterations facilitate the appearance of diabetes in already predisposed subjects, as explained in Figure 1.

If this is confirmed, attempts to stem the progression of diabetes could begin with preventive nutritional strategies, not only to decrease calorie intake but also to modulate gut microbiota with prebiotic and probiotic supplements or even through fecal transplants.

Treatment for patients who already have diabetes would also change, in that they would receive supplements to modulate the gut microbiota and improve glucose metabolism. In this regard, it would seem that some drugs already used for the treatment of diabetes, such as metformin and GLP-1RAs, are effective in lowering glycemia thanks to their action on intestinal microbiota.

To conclude, we can say that in a not too distant future, prevention and treatment for both T1D and T2D should encompass the modulation of gut microbiota and its immune responses.

## Figures and Tables

**Table 1:** Mechanisms of immunomodulation of gut microbiota in diabetes.

Animal model/study group	Main finding	Mechanisms involved	Reference
GF mice Gpr41-/- and +/+	Gpr41 is a regulator of host energy balance through modulation of gut microbiota	Reduced expression of PYY, increased intestinal transit rate, and reduced harvest of energy from the diet	B.S. Samuel et al. [34]
GF mice	Protected against obesity after consuming a Western-style, high-fat, sugar-rich diet	Elevated levels of Fiaf Increased AMPK activity	F. Backhed et al. [35]
GF mice Specific GF mice *Bacteroides fragilis* gnotobiotic mice	GF mice had a worse exercise performance compared to mice colonized by a single bacterial species and to mice colonized by multiple nonharmful bacteria	Higher serum levels of glutathione peroxidase (GPx) in SPF than GF mice. Lower serum superoxide dismutase activity in BF than SPF and GF mice	Y.J. Hsu et al. [36]
Healthy subjects	Higher fitness level is correlated to gut microbiome diversity	Increased production of butyrate	M. Estaki et al. [38]
T2D subjects	Improved glycemia, functional and anthropometric variables	Reduction of intestinal mycetes overgrowth, gut permeability, and systemic inflammation	E. Pasini et al. [39]
ob/ob mice High-fat diet-fed mice	Chronic antibiotic treatment reduced metabolic endotoxemia and the cecal content of LPS	Increased intestinal permeability Reduced expression of genes coding for proteins of tight junctions	P.D. Cani [44]
Mice injected with *Streptomyces* toxin and bafilomycin A1	Impaired glucose tolerance	Smaller islet pancreatic *β*-cell mass	M.A. Myers [46]
MyD88-negative mice NOD mice	Colonization of GF mice with intestinal bacteria reduced T1D in MyD88-negative but not in wild-type NOD mice	TLR4-mediated Trif signaling causes a tolerizing immune response	M.P. Burrows [48]
Diabetes-resistant biobreeding rats Diabetes-prone biobreeding (BBDO) rats NOD mice	Bacteria provide protection against diabetes	Transfer of intestinal *Lactobacillus johnsonii* N 6.2 from diabetes-resistant biobreeding rats to diabetes-prone biobreeding rats. Transmission of segmented filamentous bacteria to NOD mice	K. Lau et al. [51] M.A. Kriegel [52]
NOD mice placed on neutral or acidified water	Acidified water delays T1D onset	Increase in *Bacteroidetes*, *Actinobacteria*, and *Proteobacteria* and decrease in *Firmicutes* in NOD mice exposed to neutral water. Lower levels of Foxp3 expression in CD4(+)Foxp3(+) cells, decreased CD4(+)IL-17(+) cells, and a lower ratio of IL-17/IFN-*γ* CD4^+^ T-cells in NOD mice exposed to neutral water.	K.J. Wolf et al.[54]
Obese diabetic mice (wt, p40^−/−^and p35^−/−^)	Disruption of IL-12 promotes angiogenesis and increases blood flow recovery	Increase in capillary/arteriole density, endothelial nitric oxide synthase/Akt/vascular endothelial growth factor receptor 2 signaling, and a reduction in oxidative stress and inflammation	M. Ali et al. [60]
Nod2^−/−^ NOD mice Nod2^+/+^NOD mice	Nod2^−/−^ NOD mice are protected from T1D	Colonization of germ-free NOD mice with Nod2^−/−^NOD microbiota reduced the number of inflammatory cells and their cytokines, but increased T-regulatory cells	Y. Y. Li et al. [61]
Trained NOD mice Untrained NOD mice	Exercise enhances a beneficial immune-modulation in T1D	Reduced pancreatic infiltrates. Reduced levels of IL-6 and MIP-1*β*	R. Codella et. al [63]
